# Multiplex ligation reaction based on probe melting curve analysis: a pragmatic approach for the identification of 30 common *Salmonella* serovars

**DOI:** 10.1186/s12941-019-0338-5

**Published:** 2019-12-05

**Authors:** Le Zuo, Min Jiang, Yixiang Jiang, Xiaolu Shi, Yinghui Li, Yiman Lin, Yaqun Qiu, Yinhua Deng, Minxu Li, Zeren Lin, Yiqun Liao, Jianbin Xie, Qingge Li, Qinghua Hu

**Affiliations:** 1 Shenzhen Center for Disease Control and Prevention, 8 Longyuan Road, Nanshan District, Shenzhen, China; 2 College of Life Science, Sichuan University, Chengdu, China; 3 College of Life Sciences and Oceanography, Shenzhen University, Shenzhen, China; 4 School of Life Sciences, Xiamen University, Xiamen, China

**Keywords:** *Salmonella*, Serovar identification, Multiplex ligation reaction based on probe melting curve analysis, Foodborne illness

## Abstract

**Background:**

While *Salmonella* serotyping is of paramount importance for the disease intervention of salmonellosis, a fast and easy-to-operate molecular serotyping solution remains elusive. We have developed a multiplex ligation reaction based on probe melting curve analysis (MLMA) for the identification of 30 common *Salmonella* serovars.

**Methods:**

Serovar-specific primers and probes were designed based on a comparison of gene targets (*wzx* and *wzy* encoding for somatic antigen biosynthesis; *fliC* and *fljB* for flagellar antigens) from 5868 *Salmonella* genomes. The *ssaR* gene, a type III secretion system component, was included for the confirmation of *Salmonella*.

**Results:**

All gene targets were detected and gave expected Tm values during assay evaluation. Cross reactions were not demonstrated between the 30 serovars (n = 211), or with an additional 120 serovars (n = 120) and other *Enterobacteriaceae* (n = 3). The limit of identification for all targets ranged from using 1.2 ng/μL to 1.56 ng/μL of DNA. The intra- and inter-assay standard deviations and the coefficients of variation were no more than 0.5 °C and less than 1% respectively, indicating high reproducibility. From consecutive outpatient stool samples (n = 3590) collected over a 10-month period at 11 sentinel hospitals in Shenzhen, China, we conducted a multicenter study using the traditional *Salmonella* identification workflow and the MLMA assay workflow in parallel. From *Salmonella* isolates (n = 496, 13.8%) derived by both workflows, total agreement (kappa = 1.0) between the MLMA assay and conventional serotyping was demonstrated.

**Conclusions:**

With an assay time of 2.5 h, this simple assay has shown promising potential to provide rapid and high-throughput identification of *Salmonella* serovars for clinical and public health laboratories to facilitate timely surveillance of salmonellosis.

## Background

*Salmonella* is a leading cause of foodborne illness and represents a major public health burden globally. Approximately 93.8 million cases of gastroenteritis and 155,000 deaths were estimated to be caused by *Salmonella* species annually worldwide [1]. In China, *Salmonella* have been the most prevalent bacterial foodborne pathogen among diarrheal patients [2]. According to our sentinel surveillance data for infectious diarrhea, the infection rate of *Salmonella* has surpassed *Vibrio parahaemolyticus* and has become the primary cause of foodborne illnesses in Shenzhen. Currently, 2659 *Salmonella* serovars have been described on the basis of a combination of 46 somatic (O) antigens and 114 flagellar (H) antigens [3, 4]. Different *Salmonella* serovars have shown different genetic structures and host specificity, resulting in distinct epidemiology and clinical presentation, as well as differences in drug resistance and treatments [5]. For example, non-typhoidal *Salmonella* (NTS) serovars such as *S.* Typhimurium and *S.* Enteritidis have a broad host range and cause less severe and self-limiting gastroenteritis, compared to the more severe typhoid enteric fever caused by serovars Typhi and Paratyphi [6]. Certain serovars such as *S*. Choleraesuis and *S.* Dublin have been shown to cause severe systemic illness of human salmonellosis [7, 8]. Therefore, serovar identification for *Salmonella* serves a vital role in clinical diagnosis and treatment of salmonellosis, assessing trends in antibiotic resistance as well as epidemiological surveillance, outbreak detection and source attribution.

Currently, the phenotypic identification of *Salmonella* surface antigens by means of serum agglutination according to the White-Kauffmann-Le Minor scheme is regarded as the gold standard for *Salmonella* serotyping [9]. However, this conventional serological method has some important limitations, including the subjectivity in result interpretation, laborious procedures requiring highly trained personnel, slow turnaround, expensive maintenance and quality control of antisera as well as inconsistency between batches. Thus, the development of rapid molecular approaches for *Salmonella* serovar identification that exploits serovar-specific gene loci have gained continued interest over the years. Many studies have utilized multiplex PCR and quantitative PCR strategies [10–21], including those that have directly targeted O- and H- antigen-encoding genes to mirror the antigenic diversity as discerned by conventional serotyping [19–21]. Other strategies have included the use of high-resolution melting analysis [22], bead based suspension arrays [23] and pyrosequencing assay [24]. However, these methods also suffer from practical limitations, including low throughput; the ability to simultaneously identify a limited number of serovars; ambiguous results arising from gel electrophoresis; the need for supplementary PCRs for full functionality; cumbersome procedures involving the preparation of microsphere beads; and the reliance on expensive proprietary equipment and software. More recently, whole genome sequencing (WGS) data have been utilized for *Salmonella* serovar prediction in North America [25, 26], and have shown promise to replace conventional and molecular serotyping approaches in the future. However, it is currently still more costly and technically demanding, requires bioinformatics expertise and suffers from difficulties associated with the standardization and validation of methodologies [27, 28]. In contrast, simpler molecular methods are often more practical in terms of providing the serotyping information required within hours of DNA extraction.

Multiple ligation reaction based on probe melting curve (MLMA), a robust molecular typing method for the simultaneous identification of ten bacterial pathogen species [29], has been shown to reliably detect specific nucleotide sequence polymorphisms of gene targets through the combined use of oligonucleotide target probes with unique melting temperature (Tm) tags and fluorescently labelled detection probes. Based on the principles of MLMA, we developed an assay to identify 30 clinically most common *Salmonella* serovars over a 10-year period (2006–2016) in Shenzhen, China.

## Materials and methods

### Reference and clinical bacterial isolates

A total of 334 reference bacterial isolates were used for the initial development and evaluation of the MLMA assay. This comprised reference isolates (n = 211) for each of the 30 clinically most common *Salmonella* serovars observed during a 10-year period (2006–2016) from the Shenzhen Center for Disease Control and Prevention reference collection and the National Center for Medical Culture Collections of China (CMCC) (Table 1); and one reference isolate each for an additional 120 *Salmonella* serovars, *Escherichia coli*, *Shigella* and *Proteus mirabilis* for detecting cross reactions (see Additional file 1: Table S1). A total of 496 clinical isolates were obtained from a total of 3590 consecutive outpatient,stool samples in a multicenter study conducted between January to October 2017 at 11 sentinel hospitals in Shenzhen.

**Table 1:** Thirty clinically common *Salmonella* serovars (n = 211) identified by the MLMA assay

No.	Serovar	O antigen serogroup	O antigen	H1 antigen	H2 antigen	Source	Number of Isolates
1	Typhimurium	B	1,4,[5],12	i	(1,2)	CMCC 50013/SZCDC	20
2	Enteritidis	D	1,9,12	(g,m)	–	CMCC 50040/SZCDC	20
3	Paratyphi A	A	1,2,12	a	(1,5)	CMCC 50001/SZCDC	20
4	Typhi	D	9,12,[Vi]	d	–	CMCC 50097/SZCDC	10
5	Stanley	B	1,4,[5],12,[27]	d	(1,2)	SZCDC	10
6	London	E	3,[10],[15]	(l,v)	(1,6)	CMCC 50310/SZCDC	10
7	Derby	B	1,4,[5],12	(f,g)	(1,2)	SZCDC	10
8	Senftenberg	E	1,3,19	(g,s,t)	_	SZCDC	10
9	Agona	B	1,4,[5],12	(f,g,s)	(1,2)	SZCDC	10
10	Weltevreden	E	3,{10}{15}	r	z_6_	SZCDC	5
11	Anatum	E	3,{10},{15}	(e,h)	(1,6)	CMCC 50083/SZCDC	5
12	Choleraesuis	C1	6,7	c	(1,5)	SZCDC	5
13	Infantis	C1	6,7,14	r	(1,5)	CMCC 50341/SZCDC	5
14	Muenchen	C_2_-C_3_	6,8	d	(1,2)	CMCC 50125/SZCDC	5
15	Braenderup	C_1_	6,7,14	(e,h)	(e,n,z_15_)	SZCDC	5
16	Papuana	C_1_	6,7	r	(e,n,z_15_)	SZCDC	3
17	Chennai	B	4,12	d	z_35_	SZCDC	5
18	Rissen	C_1_	6,7,14	(f,g,s)	–	SZCDC	3
19	Fillmore	C_2_-C_3_	6,8	(e,h)	(e,n,x)	SZCDC	5
20	Virchow	C_1_	6,7,14	r	(1,2)	SZCDC	5
21	Saintpaul	B	1,4,[5],12	(e,h)	(1,2)	SZCDC	3
22	Litchfield	C_2_-C_3_	6,8	(l,v)	(1,2)	CMCC 50810/SZCDC	5
23	Corvallis	C_2_-C_3_	8,20	(z_4_,z_23_)	z_6_	SZCDC	5
24	Chester	B	1,4,[5],12	(e,h)	(e,n,x)	SZCDC	3
25	Heidelberg	B	1,4,[5],12	r	(1,2)	SZCDC	6
26	Give	E	3,{10}{15},{15,34}	[d].l.v	(1,7)	SZCDC	3
27	Lagos	B	1,4,[5],12	i	(1,5)	SZCDC	2
28	Essen	B	4,12	(g,m)	–	SZCDC	5
29	Nigeria	C_1_	6,7	r	(1,6)	SZCDC	5
30	Paratyphi B	B	1,4,[5],12	b	(1,2)	CMCC 50004/SZCDC	3

### Bacterial culture and conventional serotyping

Stool specimens from sentinel hospitals in the multicenter study were enriched in 10 mL selenite cystine broth (Guangdong Huankai Microbial Science & Technology, Guangzhou, China) incubated at 37 °C for 12–18 h then streaked onto BBL CHROMagar *Salmonella* agar (Bectin Dickinson, Shanghai, China) for single colonies and incubated at 37 °C for 12 h.

Suspected colonies was picked and streaked onto Triple Sugar Iron slants (Guangdong Huankai Microbial Science & Technology, Guangzhou, China) incubated at 37 °C for 12–18 h, followed by serotyping by serum agglutination based on the White Kauffman Le-Minor scheme performed according to the Chinese National Food Safety Standards: Food Microbiological Examination *Salmonella* Testing, GB 4789.4-2016.

### DNA extraction

For reference bacterial isolates, during initial development and evaluating analytical performance characteristics including the limits of identification and intra-assay and inter-assay reproducibility, DNA was extracted using QIAamp DNA Mini Kit (QIAGEN, Hilden, Germany) for accurate quantification according to manufacturer’s instructions. For clinical isolates during the multicenter study, DNA templates were prepared using the boiling method. A single colony was suspended in 50 μL of distilled water, then boiled at 100 °C for 8 min and centrifuged at 12,000×*g* for 10 min, with supernatant used as DNA template. The concentration of all DNA templates were measured spectrophotometrically (Nanodrop ND-1000, Thermo Fisher Scientific).

### Primer and probe design

The *ssaR* gene, a type III secretion system component, was incorporated into the assay as the target gene for the confirmation of *Salmonella* as previously described [30, 31]. The *wzx* and *wzy* genes encoding biosynthesis of the somatic (O) antigen; and the *fliC* and *fljB* genes encoding the phase 1 and 2 flagellar (H) antigens, were chosen as the target gene loci on the basis of their substantial antigenic diversity and heterogeneity of alleles between serovars as represented in conventional serotyping. Extensive sequence analyses were performed to identify serovar-specific polymorphisms at the target gene loci using (1) 5418 published genomes encompassing 126 *Salmonella* serovars obtained from GenBank, NCBI (http://www.ncbi.nlm.nih.gov/genbank, date accessed: March 24th 2017, see Additional file 2: Data File); and (2) 450 genomes generated (BGI, Shenzhen, China) using local *Salmonella* isolates encompassing 143 serovars from the Shenzhen Center for Disease Control isolates collection (see Additional file 2: Data File). Genomic sequence datasets were amalgamated for building an in-house database to conduct in silico sequence and phylogenetic analyses of the each targeted gene using multiple alignments (MEGA7.0) to identify polymorphic sites suitable for unequivocal differentiation of *Salmonella* serovars. Designed primers (Primer Premier v5.0) and probes (DNA folding software, online web server: http://unafold.rna.albany.edu/?q=mfold/DNA-Folding-Form) were evaluated using the BlastN algorithm (https://blast.ncbi.nlm.nih.gov/Blast.cgi). Fluorogenic probes were labeled at 5′end with reporter fluorophores carboxy fluorescein (FAM), carboxy-X-rhodamine (ROX), and indodicarbocyanine5 (Cy5) and with Black Hole Quencher sequestering molecule at the 3′ end. Subsequently, all primers and probes (see Additional file 1: Tables S2 and S3) were synthesized by Sangon Biotech (Shanghai) Co., Ltd.

### Development of the MLMA assay

Briefly, the MLMA assay consists of two key steps, (a) hybridization-ligation process followed by (b) PCR amplification and melting curve analysis. In the hybridization-ligation process, a pair of oligonucleotide probes hybridizes with the DNA template flanking either side of the target sequence, and is ligated upon complete hybridization. The ligated product is first amplified using a universal PCR primer pair via Linear After-The-Exponential (LATE) PCR, then hybridized with fluorescently labelled detection probes. *Salmonella* serovars are determined based on unique melting temperatures (Tm) obtained using a melt curve analysis established by corresponding fluorescent detection probes in the respective fluorophore channels.

The hybridization-ligation reaction was performed on a T3 Thermocycler (Biometra, Germany). Each 10 μL reaction mix contained 1.5 μL ligation probe mix (1–4 fmol of each specific ligation oligonucleotides), 1U Taq DNA ligase and 1 μL DNA ligase buffer (New England Biolabs, Bejing, China), 1.5 μL diethylpyrocarbonate (DEPC) water and 5 μL genomic DNA. Reaction conditions included an initial DNA denaturation step at 98 °C for 5 min containing only genomic DNA and ligation probe mix, a 75 °C hold to add the remainder of reaction mixture (3.5 μL), incubation step at 60 °C for 80 min, 95 °C for 5 min, and finally cooled to 4 °C. The PCR amplification and melting curve analysis was performed on a Bio-Rad CFX 96 real-time PCR system (Bio-Rad Inc., Hercules, CA). Each 50 μL reaction mix contained 1 × PCR buffer, 3 mM MgCl_2_, 2 μL of deoxynucleoside triphosphate (2.5 mM) and 1U Taq polymerase (TaKaRa Biotech Co., Dalian, China), 0.015 μM limiting primer, 0.4 μM excess primer, 0.08 μM to 0.16 μM of fluorogenic probes and 5 μL of ligation product from the hybridization-ligation reaction. Amplification consisted of an initial denaturation at 95 °C for 3 min; followed by 40 cycles of 95 °C for 5 s, 56 °C for 15 s then 74 °C for 15 s; followed by melting curve analysis starting with 2 min at 95 °C for denaturation, hybridization for 2 min at 40 °C, and a stepwise temperature increase (1 °C per 5 s) from 40 °C to 85 °C. Carboxy fluorescein (FAM), carboxy-X-rhodamine (ROX), and indodicarbocyanine5 (Cy5) fluorescence were collected and recorded.

### Analytical performance of the MLMA assay

The limit of identification was determined using a series of tenfold dilutions (from 10 to 0.01 ng) of purified DNA. The reproducibility of the assay was evaluated by using two sets of tenfold serial dilutions (3 × 10^10^ and 3 × 10^8^ copies/mL) of genomic DNA measured in triplicates.

### Multicenter study

All clinical specimens from sentinel hospitals were analyzed using the traditional *Salmonella* identification workflow and the MLMA assay workflow in parallel. Both workflows began with bacterial culture and isolation from the same stool specimen. Subsequently, the traditional identification workflow proceeded with biochemical testing and conventional serotyping; whereas the MLMA workflow proceeded with DNA extraction and MLMA assay. All procedures were performed as described in sections above. The agreement between the MLMA assay and the conventional serological method was determined by calculating the kappa value. Kappa values range from 0, indicating total disagreement, and 1, indicating total agreement of results between two methods under comparison.

## Results

### Development of the MLMA assay for the detection of antigen genes

A two-tube system, each with three fluorescence channels (ROX, FAM and Cy5) was developed (Table 2). In the first tube that detects 14 antigens, distinct Tm values in the order of high to low, the ROX channel detects *Salmonella* O antigens Group E, Group B, Group A, Group C_1_, Group C_2_–C_3_, Group D1 and the *SUC2* gene as an internal control (IC); the FAM channel detects the *Salmonella* antigens (z_4_,z_23_), *ssaR*, b, a, (f,g,s/g,s,t); whereas the Cy5 channel detects the *Salmonella* H2 antigens (1,7), (1,2), z_6_. In the second tube which detects 12 antigens, distinct Tm values in the order of high to low, the ROX channel detects *Salmonella* H antigens (e,n,x/e,n,z_15_), r, i, v, (g,m); the FAM channel detects *Salmonella* H1 antigens d, (e,h), (f,g), c; whereas the Cy5 channel detects *Salmonella* H2 antigens D2, (1,5) and (1,6). Table 3 details the experimental setup for each tube, the fluorophore channel, designated melting temperatures and corresponding antigens in the assay.

**Table 2:** Identification matrix of gene targets in a two-tube MLMA system

First tube	Antigen	Serogroup/ factors	Tm(°C)	Second tube	Antigen	Serogroup/ factors	Tm(°C)
ROX channel	O	E	74.5	ROX channel	H2	(e,n,x/e,n,z_15_)	74.0
		B	70.5		H1	r	62.5
		A	67.0			i	59.0
		C_1_	64.0			v	57.0
		C_2_-C_3_	59.0			(g,m)	54.0
		D1	54.5				
FAM channel	H1	(z_4,_z_23_)	75.5	FAM channel	H1	d	74.5
		*ssaR* b	66.0 61.0			(e,h)	64.0
		a	54.5			(f,g)	59.0
		(f,g,s/g,s,t)	50.5			c	52.5
Cy5 channel	H2	(1,7)	61.5	Cy5 channel	H2	D2	62.0
		(1,2)	55.5			(1,5)	57.0
		z6	50.5			(1,6)	50.5

**Table 3:** Determination of *Salmonella* serovars using O and H antigens identified from the two-tube MLMA system

No.	*Salmonella* serovars	O antigen	H1 antigen	H2 antigen
1	Typhimurium	1-ROX-70.5-O:4 (B)	2-ROX-59-H1-i	1-CY5-55.5-H2-(1,2)
2	Enteritidis	1-ROX-54.5-O:9 (D)	2-ROX-54-H1-(g,m)	–
3	Paratyphi A	1-ROX-67-O:2 (A)	1-FAM-54.5-H1-a	–
4	Typhi	1-ROX-54.5-O:9 (D)	2-FAM-74.5-H1-d	–
5	Stanley	1-ROX-70.5-O:4 (B)	2-FAM-74.5-H1-d	1-CY5-55.5-H2-(1,2)
6	London	1-ROX-74.5-O:3 (E)	2-ROX-57-H1-v	2-CY5-50.5-H2-(1,6)
7	Derby	1-ROX-70.5-O:4 (B)	2-FAM-59-H1-f,g	–
8	Senftenberg	1-ROX-74.5-O:3 (E)	1-FAM-50.5-H1-(f,g,s/g,s,t)	–
9	Agona	1-ROX-70.5-O:4 (B)	1-FAM-50.5-H1-(f,g,s/g,s,t)	–
10	Weltevreden	1-ROX-74.5-O:3 (E)	2-ROX-62.5-H1-r	1-CY5-50.5-H2-z_6_
11	Anatum	1-ROX-74.5-O:3 (E)	2-FAM-64-H1-(e,h)	2-CY5-50.5-H2-(1,6)
12	Choleraesuis	1-ROX-64-O:7 (C_1_)	2-FAM-52.5-H1-c	2-CY5-57-H2-(1,5)
13	Infantis	1-ROX-64-O:7 (C_1_)	2-ROX-62.5-H1-r	2-CY5-57-H2-(1,5)
14	Muenchen	1-ROX-59-O:8 (C_2_-C_3_)	2-FAM-74.5-H1-d	1-CY5-55.5-H2-(1,2)
15	Braenderup	1-ROX-64-O:7 (C_1_)	2-FAM-64-H1-(e,h)	2-ROX-74-H2-(e,n,x/e,n,z_15_)
16	Papuana	1-ROX-64-O:7 (C_1_)	2-ROX-62.5-H1-r	2-ROX-74-H2-(e,n,x/e,n,z_15_)
17	Chennai	1-ROX-70.5-O:4 (B)	2-FAM-74.5-H1-d	–
18	Rissen	1-ROX-64-O:7 (C_1_)	1-FAM-50.5-H1-(f,g,s/g,s,t)	–
19	Fillmore	1-ROX-59-O:8 (C_2_-C_3_)	2-FAM-64-H1-(e,h)	2-ROX-74-H2-(e,n,x/e,n,z_15_)
20	Virchow	1-ROX-64-O:7 (C_1_)	2-ROX-62.5-H1-r	1-CY5-55.5-H2-(1,2)
21	Saintpaul	1-ROX-70.5-O:4 (B)	2-FAM-64-H1-(e,h)	1-CY5-55.5-H2-(1,2)
22	Litchfield	1-ROX-59-O:8 (C_2_-C_3_)	2-ROX-57-H1-v	1-CY5-55.5-H2-(1,2)
23	Corvallis	1-ROX-59-O:8 (C_2_-C_3_)	1-FAM-75.5-H1-(z_4_,z_23_)	–
24	Chester	1-ROX-70.5-O:4 (B)	2-FAM-64-H1-(e,h)	2-ROX-74-H2-(e,n,x/e,n,z_15_)
25	Heidelberg	1-ROX-70.5-O:4 (B)	2-ROX-62.5-H1-r	1-CY5-55.5-H2-(1,2)
26	Give	1-ROX-74.5-O:3 (E)	2-ROX-57-H1-v	1-CY5-63.5-H2-(1,7)
27	Lagos	1-ROX-70.5-O:4 (B)	2-ROX-59-H1-i	2-CY5-57-H2-(1,5)
28	Essen	1-ROX-70.5-O:4 (B)	2-ROX-54-H1-(g,m)	–
29	Nigeria	1-ROX-64-O:7 (C_1_)	2-ROX-62.5-H1-r	2-CY5-50.5-H2-(1,6)
30	Paratyphi B	1-ROX-70.5-O:4 (B)	1-FAM-62-H1-b	1-CY5-55.5-H2-(1,2)

The table below illustrates the experimental setup for the detection of each specific antigen in the assay, including the tube number, the fluorophore channel, the designated melting temperature (Tm, °C) and the antigen being detected. As an example, the detection of O antigen in *S.* Typhimurium is represented by “1-ROX-70.5-O:4 (B)”, where “1” represents the first tube, “ROX” indicates the fluorophore channel, 70.5 indicates the melting peak temperature (°C), O:4 (B) signifies the O antigen group designation

### Performance characteristics of the MLMA assay

**Fig. 1:**
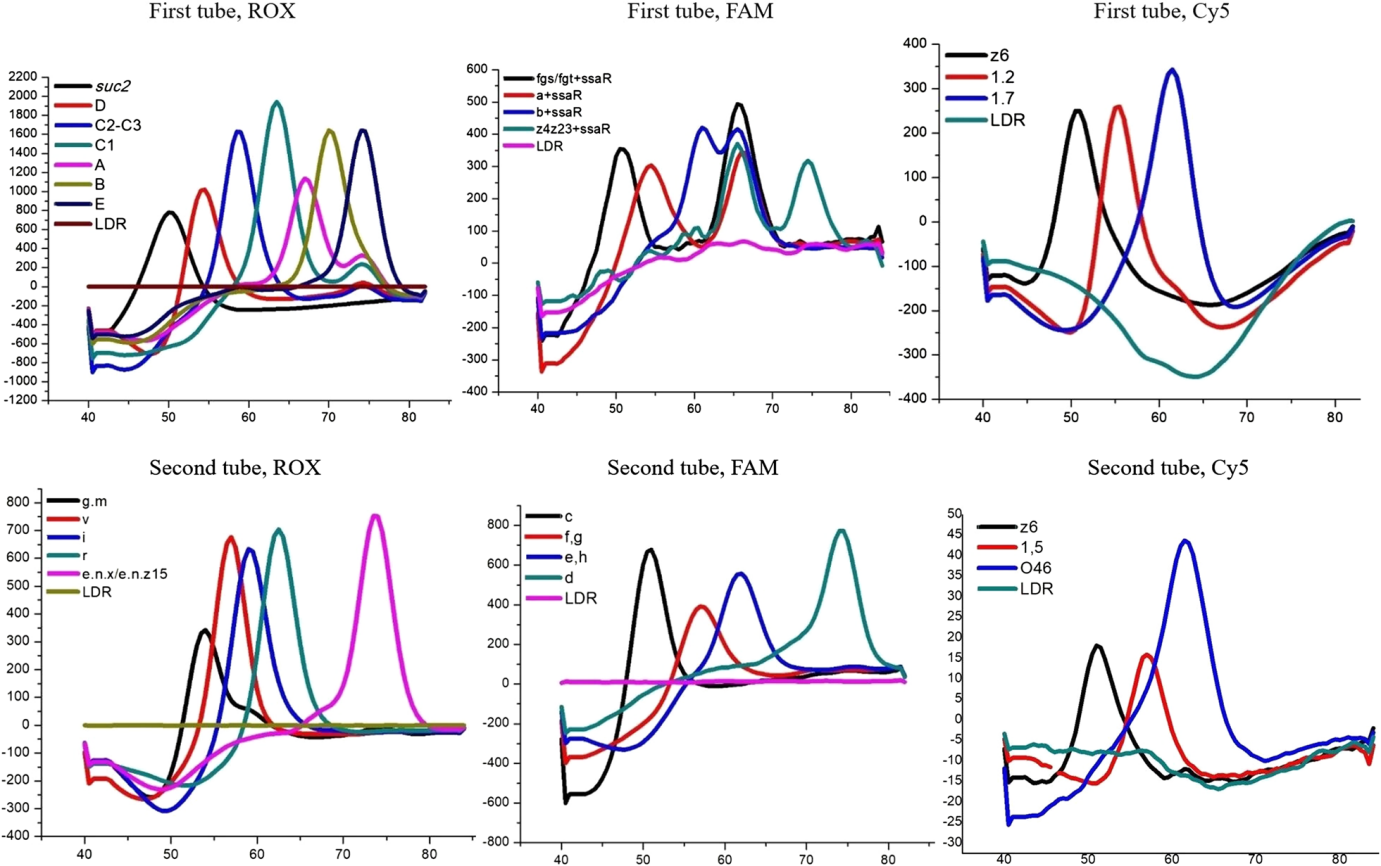
Detection of gene targets in the two-tube system for the identification of *Salmonella* and serovars by MLMA. Melting curves are color coded to represent different antigen genes in each of the three fluorophore channels (ROX, FAM, Cy5) in each of the two tubes, where the *SUC2* gene was used as a positive internal control (IC) and Ligase Detection Reaction (LDR) was used as a negative control

The MLMA assay was able to detect all gene targets and gave expected Tm values for each serovar (Fig. 1.). Cross reactions were not detected between the 30 serovars (n = 211), or with an additional *Salmonella* serovars (n = 120), *Escherichia coli* (n = 1), *Shigella* (n = 1) and *Proteus mirabilis* (n = 1) (see Additional file 1: Table S1). Due to the inaccessibility of certain rare serovars with closely related antigenic formula, potential cross reactions with these rare clinical strains could not be tested, as detailed in discussion. The limit of identification ranged from 1.2 to 1.56 ng/μL of genomic DNA concentration for each serovar (see Additional file 1: Table S4). The standard deviations (SD) and the coefficient of variation (CV) for intra-assay and inter-assay reproducibility were calculated (see Additional file 1: Table S5). The largest SD value for mean Tm was no more than 0.5 °C, whereas the CV were less than 1% for both intra-assay and inter-assay reproducibility analyses.

### Multicenter study

From a total of 3590 consecutive outpatient stool samples collected, 496 clinical *Salmonella* isolates (13.8%) were identified by both the traditional identification and MLMA workflows. Among these, 383 isolates (77.2%) belonged to the 29 of the 30 *Salmonella* serovars identifiable by the MLMA workflow, whereas 113 isolates (22.8%) belonged to 27 other *Salmonella* serovars. The MLMA results demonstrated kappa values of 1.0 for each of the serovars identified when compared against conventional serotyping results. A full comparison of serovar identification by MLMA and conventional serotyping for all clinical isolates is available (see Additional file 1: Table S6). In the clinical setting, the MLMA assay workflow was 12–18 h faster compared to traditional workflow, with a total assay time of 2.5 h to *Salmonella* serotyping results from DNA extraction.

## Discussion

*Salmonella* infection remains a significant public health challenge worldwide, and given the paramount importance of serotyping as a crucial tool for epidemiological investigations and surveillance of salmonellosis, there is a continued need for the development of a rapid and accurate molecular serotyping method that is also simple in operation and economical for high-throughput application. In this study, we have developed a multiple ligation reaction based on probe melting curve analysis (MLMA) [29] for the identification of clinically common *Salmonella* serovars.

The newly developed assay was able to detect all gene targets were and gave expected Tm values with robust performance characteristics. The assay has demonstrated high reproducibility where the largest SD value for mean Tm was no more than 0.5 °C and the CVs were less than 1% in both intra-assay and inter-assay reproducibility analyses; whereas the detection limit for the 30 *Salmonella* serovars ranged from 1.2 to 1.56 ng/μL. Overall, these performance characteristics are similar to those observed in an assay developed for the rapid identification of foodborne pathogens [29]. In the multicenter study we conducted, the MLMA assay has shown total concordance with the conventional serological method. Serovars for all of the isolates (77.2%) covered by the assay were correctly identified and no false positive identification of non-target serovars was observed, as indicated by kappa values of 1.0 for each of the *Salmonella* serovars identified. This can be attributed to the high fidelity of the DNA ligase to recognize gaps between nucleic acid sequences, and that the oligonucleotide probes must be completely hybridized with the specific flanking nucleotides of the target sequence for ligation to occur. The MLMA assay workflow presented here was 12–18 h faster compared to the traditional workflow, which required overnight incubation for biochemical identification tests prior to conventional serotyping. From DNA extraction, the assay consists only of two thermocycling sets, with minimal hands-on time and a total assay time of approximately 2.5 h to both *Salmonella* confirmation and serotyping results at once. Therefore, the current assay presents a prospective method for the simple and rapid detection of *Salmonella* and serovar identification.

In developing this assay, we included 30 clinically most common *Salmonella* serovars isolated (87.9%) from human infections over a 10-year period (2006–2016), in Shenzhen, China for rapid identification. Incidentally, these serovars also covered 72.8% of the most common serovars isolated from diarrheal patients domestically in China [32]; whereas internationally, these serovars were well represented among the 20 most frequently identified clinical isolates for countries in Asia (n = 14), Europe (n = 11), North America (n = 11), Latin America (n = 12) and Oceania (n = 10) [33].

In recent years, various molecular approaches have been investigated and developed for the identification of *Salmonella* serovars to circumvent the limitations of conventional serotyping. Among these, multiplex PCRs and quantitative PCRs have proved to be popular methods [10–21]. Other methods included the use of the Bio-Plex system and adapting the Luminex platform, a DNA sensor-based suspension array for the identification of eight common *Salmonella* serovars was developed with a total assay time of 3.5 h [23]. A PCR-pyrosequencing assay has also been exploited as a rapid serotyping method for seven most common serovars in Canada [24]. However, the application of these methods has a number of limitations, including the few number of serovars that could be identified simultaneously; the need for gel electrophoresis; the reliance on expensive proprietary molecular biology equipment and analyzers; time consuming protocols such as the coupling of oligonucleotide probes to microsphere beads; the demand for sequence analysis knowledge and software. A high-resolution melting analysis (HRM) has also been described using arbitrary melting curve profiles found in several surrogate genomic markers to index and differentiate *Salmonella* serovars [22]. However, this methodology resulted in identical melting curve profiles found for different serovars and requires a separate PCR assay for full functionality. In contrast, the MLMA methodology is fundamentally different by the combined use of serovar-specific oligonucleotide target probes with designated Tm tags and fluorescently labelled detection probes, resulting in a highly robust and reliable assay.

In the current assay, we selected the genes encoding for the somatic (O) and the flagellar (H) antigen as our target genes for identification of *Salmonella* serovars because they are directly correlated with the White-Kaufmann-Le Minor scheme. This allows direct comparison with conventional serotyping results and has advantage over indirect approaches that relies on surrogate molecular markers approximated to be serovar-specific for the prediction or correlation of serovars. The selection of the *Salmonella* serovar surface antigen genes are highly crucial in order to ensure sufficient genetic diversity to avoid cross reaction with other *Salmonella* serovars or other bacterial species. The *rfb* gene regulon contain a cluster of genes required for O antigen biosynthesis and assembly, thereby responsible for the substantial antigenic diversity of the *Salmonella* O antigens that is exploited for serogroup identification in conventional serotyping [34]. In particular, the O antigen transporter (*wzx*) and O antigen polymerase (*wzy*) [35] have been chosen as an ideal target for the current assay as they share little sequence homology between different O antigen serogroups even at the amino acid level [36]. Similarly, it has been shown that the *fliC* and *fljB* genes encoding for the phase 1 and phase 2 flagellar (H) antigens in *Salmonella* are sufficiently heterologous among different alleles but remained homologous between the same alleles [37], making it a practical choice of target in our assay. Based on the current number of O and H antigens detected in the assay for the common serovars, the combination of these antigens could potentially identify additional serovars. For example, we conducted additional tests using the assay to successfully identify serovars such as *Salmonella* Hidalgo O antigen O: 8 (C_2_–C_3_) group. This shows that the current system has the potential to detect additional *Salmonella* serovar antigens and is amenable to modifications for increasing the number of serovars as we plan to expand the current assay to include up to 100 serovars from worldwide identified in the future.

There are several limitations to our study. In the current assay configuration, certain rare serovars with closely related antigenic formula containing *Salmonella* antigens/complexes E1/E4; f,g,s/g,[s],t and e,n,x/e,n,z15 may not be sufficiently discerned and hence the possibility for cross-reaction with the 30 common serovars. These include *S*. Senftenburg with *S*. Bloomsbury II; *S*. Agona with *S*. Budapest, *S*. Anatum with *S*. Hayindogo, *S*. Cholerasuis with *S*. Paratyphi C and *S*. Typhisuis; *S*. Chennai and *S*. Mons; *S*. Fillmore with *S*. Tshiongwe; *S*. Chester with *S*. Sandiego; and *S*. Give with *S*. Parkroyal. Although these serovars are rare, further development of the assay would still be required to address these limitations and the acquisition of rare isolates at our laboratory would be essential.

There are a number of aspects in which the current assay could be further improved. The detection throughput could be further increased by adding more melting point tags and using additional fluorophore channels. A total of 26 genes are detected using three fluorophore channels in the current assay configuration. Theoretically however, each fluorophore channel could carry six and up to eight Tm tags, which we have achieved experimentally using the ROX channel (data not shown). Accordingly, our assay could potentially be further enhanced to detect up to 32 genes by using four fluorophore channels which may in turn identify additional *Salmonella* serovars. However, as the number of genes detected is increased, we anticipate greater difficulties to establish a stable assay considering the influence of additional probes are introduced. Another useful aspect worth exploring would be its application to clinical and food samples by direct amplification of target genes by incorporating inhibitor resistant polymerases into the assay to avoid the tedious process of isolation and DNA extraction from culture.

In recent years, the rapid advancements of whole genome sequencing (WGS) technologies has facilitated an expansion of important studies of genomic epidemiology [38], population structure [39] and microbial evolution [40] for *Salmonella*, and has emerged as an alternative to replace current molecular subtyping methods [25, 26]. However, gaps still exists for routine use of WGS due to cost, consistent standards and bioinformatics resources, which remains impractical and challenging for most public health laboratories, especially for low and middle income countries with less developed public health infrastructure [27, 28]. Indeed, we have already embarked on developing WGS-based methods with preliminary success; the very impetus however behind developing the current assay was to bridge the gap before WGS would become sufficiently ready to gain widespread acceptance and adoption in the public health laboratories in China.

## Conclusions

In this study, we have developed and demonstrated the promising utility of an MLMA assay for the identification of clinically most common *Salmonella* serovars. Further development to the assay by increasing the number of serovars for identification and addressing the current limitations will be our research priority. We have shown that by using relatively basic molecular biology procedures and equipment, the MLMA methodology has great potential to serve as a pragmatic and inexpensive solution for the timely and effective public health intervention of salmonellosis.

## Supplementary information

**Additional file 1: Table S1.** Additional *Salmonella* serovars (n = 120) and other *Enterobacteriaceae* (n = 3) used for assessing the specificity of MLMA assay. **Table S2.** Hybridization oligonucleotides probe sequences for specific *Salmonella* O and H antigens and the *ssaR* gene. **Table S3.** Sequence of universal primers for LATE-PCR and fluorescent detection probes for melting curve analysis. **Table S4.** Limit of identification values of each *Salmonella* serovar in the MLMA assay. **Table S5.** Reproducibility of designated melting temperature (Tm) tags for each gene target in the MLMA assay. **Table S6.** *Salmonella* serovar identification by MLMA assay and conventional serotyping among clinical isolates (n = 383) from the multicenter study.

**Additional file 2: Data File.** Local and public *Salmonella* genomes (n = 5868) used for sequence analyses for identification of serovar-specific polymorphisms.

## Data Availability

All data generated or analyzed during this study are included in this published article and additonal files.

## Abbreviations

MLMA: multiplex ligation reaction based on probe melting curve analysis
NTS: non-typhoidal *Salmonella*
Tm: melting temperature
FAM: carboxy fluorescein
ROX: carboxy-X-rhodamine
Cy5: indodicarbocyanine5
LATE PCR: linear after-the-exponential PCR
DEPC: diethylpyrocarbonate
IC: internal control

## References

[1] Majowicz SE, Musto J, Scallan E, Angulo FJ, Kirk M, O’Brien SJ, Jones TF, Fazil A, Hoekstra RM (2010). The global burden of nontyphoidal *Salmonella gastroenteritis*. Clin Infect Dis.

[2] Liu J, Bai L, Li W, Han H, Fu P, Ma X, Bi Z, Yang X, Zhang X, Zhen S (2018). Trends of foodborne diseases in China: lessons from laboratory-based surveillance since 2011. Front Med.

[3] Guibourdenche M, Roggentin P, Mikoleit M, Fields PI, Bockemuhl J, Grimont PA, Weill FX (2010). Supplement 2003–2007 (No. 47) to the White-Kauffmann-Le Minor scheme. Res Microbiol.

[4] Issenhuth-Jeanjean S, Roggentin P, Mikoleit M, Guibourdenche M, de Pinna E, Nair S, Fields PI, Weill FX (2014). Supplement 2008–2010 (no 48) to the White-Kauffmann-Le Minor scheme. Res Microbiol.

[5] Fierer J, Guiney DG (2001). Diverse virulence traits underlying different clinical outcomes of *Salmonella* infection. J Clin Investig.

[6] Langridge GC, Nair S, Wain J (2009). Nontyphoidal *Salmonella* serovars cause different degrees of invasive disease globally. J Infect Dis.

[7] Chen PL, Wu CJ, Chang CM, Lee HC, Lee NY, Shih HI, Lee CC, Ko NY, Wang LR, Ko WC (2007). Extraintestinal focal infections in adults with *Salmonella enterica* serotype Choleraesuis bacteremia. J Microbiol Immunol Infect.

[8] Harvey RR, Friedman CR, Crim SM, Judd M, Barrett KA, Tolar B, Folster JP, Griffin PM, Brown AC (2017). Epidemiology of *Salmonella enterica* serotype dublin infections among humans, United States, 1968–2013. Emerg Infect Dis.

[9] Grimont PAD, Weill FX (2007). Antigenic formulae of the *Salmonella* serovars.

[10] Akiba M, Kusumoto M, Iwata T (2011). Rapid identification of *Salmonella enterica* serovars, Typhimurium, Choleraesuis, Infantis, Hadar, Enteritidis, Dublin and Gallinarum, by multiplex PCR. J Microbiol Methods.

[11] Barco L, Lettini AA, Ramon E, Longo A, Saccardin C, Pozza MC, Ricci A (2011). A rapid and sensitive method to identify and differentiate *Salmonella enterica* serotype Typhimurium and *Salmonella enterica* serotype 4,[5],12:i:- by combining traditional serotyping and multiplex polymerase chain reaction. Foodborne Pathog Dis.

[12] Bugarel M, Tudor A, Loneragan GH, Nightingale KK (2017). Molecular detection assay of five *Salmonella* serotypes of public interest: Typhimurium, Enteritidis, Newport, Heidelberg, and Hadar. J Microbiol Methods.

[13] Heymans R, Vila A, van Heerwaarden CAM, Jansen CCC, Castelijn GAA, van der Voort M, Biesta-Peters EG (2018). Rapid detection and differentiation of *Salmonella* species, *Salmonella* Typhimurium and *Salmonella* Enteritidis by multiplex quantitative PCR. PLoS ONE.

[14] Li R, Wang Y, Shen J, Wu C (2014). Development of a novel hexa-plex PCR method for identification and serotyping of *Salmonella* species. Foodborne Pathog Dis.

[15] Munoz N, Diaz-Osorio M, Moreno J, Sanchez-Jimenez M, Cardona-Castro N (2010). Development and evaluation of a multiplex real-time polymerase chain reaction procedure to clinically type prevalent *Salmonella enterica* serovars. J Mol Diagn.

[16] Park SH, Ricke SC (2015). Development of multiplex PCR assay for simultaneous detection of *Salmonella* genus, *Salmonella* subspecies I, *Salm. Enteritidis*, *Salm. Heidelberg* and *Salm. Typhimurium*. J Appl Microbiol.

[17] Prendergast DM, Hand D, Niota Ghallchoir E, McCabe E, Fanning S, Griffin M, Egan J, Gutierrez M (2013). A multiplex real-time PCR assay for the identification and differentiation of *Salmonella enterica* serovar Typhimurium and monophasic serovar 4,[5],12:i. Int J Food Microbiol.

[18] Xiong D, Song L, Pan Z, Jiao X (2018). Identification and discrimination of *Salmonella enterica* Serovar Gallinarum Biovars Pullorum and Gallinarum based on a one-step multiplex PCR assay. Front Microbiol.

[19] Cardona-Castro N, Sanchez-Jimenez M, Lavalett L, Munoz N, Moreno J (2009). Development and evaluation of a multiplex polymerase chain reaction assay to identify *Salmonella* serogroups and serotypes. Diagn Microbiol Infect Dis.

[20] Herrera-Leon S, Ramiro R, Arroyo M, Diez R, Usera MA, Echeita MA (2007). Blind comparison of traditional serotyping with three multiplex PCRs for the identification of *Salmonella* serotypes. Res Microbiol.

[21] Hong Y, Liu T, Lee MD, Hofacre CL, Maier M, White DG, Ayers S, Wang L, Berghaus R, Maurer JJ (2008). Rapid screening of *Salmonella enterica* serovars Enteritidis, Hadar, Heidelberg and Typhimurium using a serologically-correlative allelotyping PCR targeting the O and H antigen alleles. BMC Microbiol.

[22] Zeinzinger J, Pietzka AT, Stoger A, Kornschober C, Kunert R, Allerberger F, Mach R, Ruppitsch W (2012). One-step triplex high-resolution melting analysis for rapid identification and simultaneous subtyping of frequently isolated Salmonella serovars. Appl Environ Microbiol.

[23] Aydin M, Carter-Conger J, Gao N, Gilmore DF, Ricke SC, Ahn S (2018). Molecular identification of common *Salmonella* serovars using multiplex DNA sensor-based suspension array. Anal Bioanal Chem.

[24] Furukawa M, Goji N, Janzen TW, Thomas MC, Ogunremi D, Blais B, Misawa N, Amoako KK (2018). Rapid detection and serovar identification of common *Salmonella enterica* serovars in Canada using a new pyrosequencing assay. Can J Microbiol.

[25] Zhang S, Yin Y, Jones MB, Zhang Z, Deatherage Kaiser BL, Dinsmore BA, Fitzgerald C, Fields PI, Deng X (2015). Salmonella serotype determination utilizing high-throughput genome sequencing data. J Clin Microbiol.

[26] Yoshida CE, Kruczkiewicz P, Laing CR, Lingohr EJ, Gannon VP, Nash JH, Taboada EN (2016). The *Salmonella* in silico typing resource (SISTR): an open web-accessible tool for rapidly typing and subtyping draft *Salmonella* genome assemblies. PLoS ONE.

[27] WorldHealthOrganization. Whole genome sequencing for foodborne disease surveillance; 2018. http://www.whoint/foodsafety/publications/foodborne_disease/wgs_landscape/en/. Accessed 12 Jul 2019.

[28] Bogaerts B, Winand R, Fu Q, Van Braekel J, Ceyssens PJ, Mattheus W, Bertrand S, De Keersmaecker SCJ, Roosens NHC, Vanneste K (2019). Validation of a bioinformatics workflow for routine analysis of whole-genome sequencing data and related challenges for pathogen typing in a European National Reference Center: *Neisseria meningitidis* as a proof-of-concept. Front Microbiol.

[29] Jiang Y, He L, Wu P, Shi X, Jiang M, Li Y, Lin Y, Qiu Y, Bai F, Liao Y (2017). Simultaneous identification of ten bacterial pathogens using the multiplex ligation reaction based on the probe melting curve analysis. Sci Rep.

[30] Hu Q, Coburn B, Deng W, Li Y, Shi X, Lan Q, Wang B, Coombes BK, Finlay BB (2008). *Salmonella enterica* serovar Senftenberg human clinical isolates lacking SPI-1. J Clin Microbiol.

[31] Hu Q, Lyu D, Shi X, Jiang Y, Lin Y, Li Y, Qiu Y, He L, Zhang R, Li Q (2014). A modified molecular beacons-based multiplex real-time PCR assay for simultaneous detection of eight foodborne pathogens in a single reaction and its application. Foodborne Pathog Dis.

[32] Ran L, Wu S, Gao Y, Zhang X, Feng Z, Wang Z, Kan B, Klena JD, Lo FO, Wong DM, Angulo FJ (2011). Laboratory-based surveillance of nontyphoidal *Salmonella* infections in China. Foodborne Pathog Dis.

[33] Hendriksen RS, Vieira AR, Karlsmose S, Lo F, Wong DM, Jensen AB, Wegener HC, Aarestrup FM (2011). Global monitoring of *Salmonella* serovar distribution from the World Health Organization Global Foodborne Infections Network Country Data Bank: results of quality assured laboratories from 2001 to 2007. Foodborne Pathog Dis.

[34] Xiang SH, Haase AM, Reeves PR (1993). Variation of the rfb gene clusters in *Salmonella enterica*. J Bacteriol.

[35] Samuel G, Reeves P (2003). Biosynthesis of *O*-antigens: genes and pathways involved in nucleotide sugar precursor synthesis and O-antigen assembly. Carbohyd Res.

[36] Fitzgerald C, Sherwood R, Gheesling LL, Brenner FW, Fields PI (2003). Molecular analysis of the rfb O antigen gene cluster of *Salmonella enterica* serogroup O:6,14 and development of a serogroup-specific PCR assay. Appl Environ Microbiol.

[37] McQuiston JR, Parrenas R, Ortiz-Rivera M, Gheesling L, Brenner F, Fields PI (2004). Sequencing and comparative analysis of flagellin genes fliC, fljB, and flpA from *Salmonella*. J Clin Microbiol.

[38] Deng X, Desai PT, den Bakker HC, Mikoleit M, Tolar B, Trees E, Hendriksen RS, Frye JG, Porwollik S, Weimer BC (2014). Genomic epidemiology of *Salmonella enterica* serotype Enteritidis based on population structure of prevalent lineages. Emerg Infect Dis.

[39] Alikhan NF, Zhou Z, Sergeant MJ, Achtman M (2018). A genomic overview of the population structure of *Salmonella*. PLoS Genet.

[40] Langridge GC, Fookes M, Connor TR, Feltwell T, Feasey N, Parsons BN, Seth-Smith HM, Barquist L, Stedman A, Humphrey T (2015). Patterns of genome evolution that have accompanied host adaptation in *Salmonella*. Proc Natl Acad Sci USA.

