# Supplementary material for: Multiplex ligation reaction based on probe melting curve analysis: a pragmatic approach for the identification of 30 common Salmonella serovars
Source: Ann Clin Microbiol Antimicrob. 2019 Dec 5;18:39. doi: 10.1186/s12941-019-0338-5 (PMC6894471; doi:10.1186/s12941-019-0338-5)
Supplement: Supplementary file 1 — Additional file 1: Table S1. Additional Salmonella serovars (n = 120) and other Enterobacteriaceae (n = 3) used for assessing the specificity of MLMA assay. Table S2. Hybridization oligonucleotides probe sequences for specific Salmonella O and H antigens and the ssaR gene. Table S3. Sequence of universal primers for LATE-PCR and fluorescent detection probes for melting curve analysis. Table S4. Limit of identification values of each Salmonella serovar in the MLMA assay. Table S5. Reproducibility of designated melting temperature (Tm) tags for each gene target in the MLMA assay. Table S6. Salmonella serovar identification by MLMA assay and conventional serotyping among clinical isolates (n = 383) from the multicenter study. [file 12941_2019_338_MOESM1_ESM.docx]

**SUPPLEMENTAL TABLES**

Table S1. Additional *Salmonella* serovars (n=120) and other *Enterobacteriaceae* (n=3) used for assessing the specificity of MLMA assay

| No. | Serovar | O antigen | O antigen | H1 | H2 | Source |
| --- | --- | --- | --- | --- | --- | --- |
|  |  | serogroup |  | antigen | antigen |  |
| 1 | Bovismorbificans | C_2_-C_3_ | 6,8,20 | V,[i] | 1,5 | SZCDC |
| 2 | Newlands | E1 | 3,[10][15.34] | e,h | e.n,x | SZCDC |
| 3 | Newport | C_2_-C_3_ | 6,8,20 | e,h | e,n,x | SZCDC |
| 4 | Lomita | C_1_ | 6,7 | e,h | 1,5 | SZCDC |
| 5 | Stanleyville | B | 1,4，[5]，12,[27] | z4,z23 | [1,2] | SZCDC |
| 6 | Brezany | B | 1,4,12,27 | d | 1,6 | SZCDC |
| 7 | Hidalgo | C_2_-C_3_ | 6,8 | y,[i] | e,n,z15 | SZCDC |
| 8 | Ughelli | E1 | 3,10 | r | 1,5 | SZCDC |
| 9 | Istanbul | C_2_-C_3_ | 8 | z10 | e,n,x | SZCDC |
| 10 | Albany | C_2_-C_3_ | 8,20 | z4,z24 | - | SZCDC |
| 11 | Amherstiana | C_2_-C_3_ | 8 | l,v | 1,6 | SZCDC |
| 12 | Bsilla | C_2_-C_3_ | 6,8 | r | 1,2 | SZCDC |
| 13 | Sarajane | B | 1,4,[5],12,[27] | d | e,n,x | SZCDC |
| 14 | Altona | C_2_-C_3_ | 8,20 | r,[i] | z6 | SZCDC |
| 15 | Hindmarsh | C_2_-C_3_ | 8,20 | r | 1,5 | SZCDC |
| 16 | holcomb | C_2_-C_3_ | 6,8 | L,V | e,n,x | SZCDC |
| 17 | Blegdam | D1 | 9,12 | g,m,q | - | SZCDC |
| 18 | Hato II | B | 1,4,12,[27] | g,m.t | z39 | SZCDC |
| 19 | Kottbus | C_2_-C_3_ | 6,8 | e,h | 1,5 | SZCDC |
| 20 | Nchange | E1 | 3,{10}{15} | l,v | 1,2 | SZCDC |
| 21 | Ruzizi | E1 | 3,10 | l,v | z6 | SZCDC |
| 22 | Seremban | D1 | 9,12 | i | 1,5 | SZCDC |
| 23 | Bareilly | C_1_ | 6,7,14 | y | 1,5 | SZCDC |
| 24 | Eingedi | C_1_ | 6,7 | f,g,t | 1,2,7 | SZCDC |
| 25 | Gueuletapee | D1 | 9,12 | g,m,s | - | SZCDC |
| 26 | Kentucky | C_2_-C_3_ | 8,20 | 2 | z6 | SZCDC |
| 27 | Mkamba | C_1_ | 6,7 | l,v | 1,6 | SZCDC |
| 28 | Norwich | C_1_ | 6,7 | e,h | 1,6 | SZCDC |
| 29 | Oranienburg | C_1_ | 6,7,14 | m,t | [z57] | SZCDC |
| 30 | Schwarzengrund | B | 1,4,12,27 | d | 1,7 | SZCDC |
| 31 | Singapore | C_1_ | 6,7 | k | e,n,x | SZCDC |
| 32 | Bardo | C_2_-C_3_ | 8 | e,h | 1,2 | SZCDC |
| 33 | Hadar | C_2_-C_3_ | 6,8 | z10 | e,n,x | SZCDC |
| 34 | Hartford | C_1_ | 6,7 | y | e,n,x | SZCDC |
| 35 | Irummu | C_1_ | 6,7 | l,v | 1,5 | SZCDC |
| 36 | Jerusalem | C_1_ | 6,7,14 | Z10 | 1,w | SZCDC |
| 37 | Kolda | C_2_-C_3_ | 8,20 | z35 | 1,2 | SZCDC |
| 38 | Lika | C_1_ | 6,7 | i | 1,7 | SZCDC |
| 39 | Manchester | C_2_-C_3_ | 6,8 | l,v | 1,7 | SZCDC |
| 40 | Meleagridis | E1 | 3,[10],[15] |  |  | SZCDC |
| 41 | Montevideo | C1 | {6,7,14},{54} | g,m,[p],s | [1.2.7] | SZCDC |
| 42 | Nitra | A | 2,12 | g,m | - | SZCDC |
| 43 | Paratyphi C | C1 | 6,7,[Vi] | c | 1,5 | SZCDC |
| 44 | Regent | E1 | 3,10 | f,g,[s] | [1,6] | SZCDC |
| 45 | Simi | E1 | 3,10 | r | e,n,z15 | SZCDC |
| 46 | Sinchew | E1 | 3,10 | l,v | z35 | SZCDC |
| 47 | Sinstorf | E1 | 3,10 | l,v | 1,5 | SZCDC |
| 48 | Thompson | C1 | 6,7,14 | k | 1,5 | SZCDC |
| 49 | Winneba | B | 4,12 | r | 1,6 | SZCDC |
| 50 | Akanji | C_2_-C_3_ | 6,8 | R | 1,7 | SZCDC |
| 51 | Altendorf | B | 4,12,[27] | c | 1,7 | SZCDC |
| 52 | Amounderness | E1 | 3,10 | i | 1,5 | SZCDC |
| 53 | Angers | C_2_-C_3_ | 8,20 | z35 | z6 | SZCDC |
| 54 | Bonariensis | C_2_-C_3_ | 6,8 | i | e,n,x | SZCDC |
| 55 | Bonn | C_1_ | 6,7 | 1，v | e,n,x | SZCDC |
| 56 | Borbeck | G | 13,22 | 1,v | 1,6 | SZCDC |
| 57 | Bredeney | B | 1,4,12,27 | l,v | 1,7 | SZCDC |
| 58 | Bury | B | 4,12,27 | c | z6 | SZCDC |
| 59 | Cerro | H | 6,14,18 | z4,z23 | [1,5] | SZCDC |
| 60 | Chincol | C_2_-C_3_ | 6,8 | g,m,[s] | [e,n,x] | SZCDC |
| 61 | Djugu | C_1_ | 6,7 | z10 | e,n,x | SZCDC |
| 62 | Dublin | D1 | 1,9,12,[Vi] | g,p | - | SZCDC |
| 63 | Fulda | E4 | 1,3,19 | 1,w | 1,5 | SZCDC |
| 64 | Houston | D1 | 9,12 | l,v | d,1,5 | SZCDC |
| 65 | Indiana | B | 1,4,12 | z | 1,7 | SZCDC |
| 66 | Israel | D1 | 9,12 | e,h | e,n,z15 | SZCDC |
| 67 | Ivory | I | 16 | r | 1,6 | SZCDC |
| 68 | Jamaica | D1 | 9,12 | r | 1,5 | SZCDC |
| 69 | Kingston | B | 1,4,[5],12,[27] | g,s,t | [1,2] | SZCDC |
| 70 | Larochelle | C_1_ | 6,7 | e,h | 1,2 | SZCDC |
| 71 | Menston | C_1_ | 6,7 | g,s,[t] | [1,6] | SZCDC |
| 72 | Missouri | F | 11 | g,s,t | - | SZCDC |
| 73 | Muenster | E1 | 3,{10}{15}{15,34} | e,h | 1,5 | SZCDC |
| 74 | Nagoya | C_2_-C_3_ | 6,8 | b | 1,5 | SZCDC |
| 75 | Newrochelle | E1 | 3,10 | k | l,w | SZCDC |
| 76 | Omuna | C_1_ | 6,7 | z10 | z35 | SZCDC |
| 77 | Othmarschen | C_1_ | 6,7,14 | g,m,[t] | - | SZCDC |
| 78 | Pakistan | C_2_-C_3_ | 8 | l,v | 1,2 | SZCDC |
| 79 | Pasing | B | 4,12 | z35 | 1,5 | SZCDC |
| 80 | Poona | G | 1,13,22 | z | 1,6 | SZCDC |
| 81 | Potsdam | C_1_ | 6,7,14 | l,v | z6 | SZCDC |
| 82 | Reading | B | 1,4,[5],12 | e,h | 1,2 | SZCDC |
| 83 | Rechovot | C_2_-C_3_ | 8,20 | e,h | z6 | SZCDC |
| 84 | Richmond | C_1_ | 6,7 | y | 1,2 | SZCDC |
| 85 | Rissen | C_1_ | 6,7,14 | f,g,[s] | - | SZCDC |
| 86 | Seegefeld | E1 | 3,10 | r,i | 1,2 | SZCDC |
| 87 | Senegal | F | 11 | r | 1,5 | SZCDC |
| 88 | Seremban | D1 | 9,12 | i | 1,5 | SZCDC |
| 89 | Tallahassee | C_2_-C_3_ | 6,8 | z4,z32 | - | SZCDC |
| 90 | Tilburg | E4 | 1,3,19 | d | 1,w | SZCDC |
| 91 | Tumodi | B | 1,4,12 | i | z6 | SZCDC |
| 92 | Uganda | E1 | 3,{10}{15} | 1.z13 | 1,5 | SZCDC |
| 93 | Vejle | E1 | 3,{10}{15} | e,h | 1,2 | SZCDC |
| 94 | Umbadah | E4 | 1,3,19 | d | 1,2 | SZCDC |
| 95 | Wanatah | E4 | 1,3,19 | d | 1,7 | SZCDC |
| 96 | Weneziana | F | 11 | i | e,n,x | SZCDC |
| 97 | Weybridge | E1 | 3,10 | d | z6 | SZCDC |
| 98 | Yaounde | B | 1,4,12,27 | z35 | e,n,z15 | SZCDC |
| 99 | Yovokome | C_2_-C_3_ | 8,20 | d | 1,5 | SZCDC |
| 100 | Dessau | E1 | 1,3,15,19 | g,s,t | - | SZCDC |
| 101 | Tennessee | C_1_ | 6,7,14 | z29 | [1,2,7] | SZCDC |
| 102 | Rubislaw | F | 11 | r | e,n,x | SZCDC |
| 103 | Javiana | D1 | 1,9,12 | 1,z28 | 1,5 | SZCDC |
| 104 | Mbandaka | C_1_ | 6,7,14 | z10 | e,n,z15 | SZCDC |
| 105 | Gallinarum | D1 | 1,9,12 | __ | __ | SZCDC |
| 106 | Inverness | P | 38 | k | 1,6 | SZCDC |
| 107 | Berta | C_1_ | 6,7,14 | y | 1,5 | SZCDC |
| 108 | Pullorum | D1 | 1,9,12 | __ | __ | SZCDC |
| 109 | Worthington | G | 1,13,22 | z | 1,w | SZCDC |
| 110 | Johannesburg | R | 1,40 | b | e,n,x | SZCDC |
| 111 | Cubana | G | 1,13,23 | z29 | __ | SZCDC |
| 112 | Brandenburg | B | 4,[5] ,12 | 1,v | e,n,z15 | SZCDC |
| 113 | Gaminara | I | 16 | d | 1,7 | SZCDC |
| 114 | Manhattan | C_2_-C_3_ | 6,8 | d | 1,5 | SZCDC |
| 115 | Minnesota | L | 21 | b | e,n,x | SZCDC |
| 116 | Ohio | C_1_ | 6,7,14 | b | 1,w | SZCDC |
| 117 | Hvittingfoss | I | 16 | b | e,n,x | SZCDC |
| 118 | Liverpool | E4 | 1,3,19 | d | e,n,z15 | SZCDC |
| 119 | Livingstone | C1 | 6,7,14 | d | 1,w | SZCDC |
| 120 | Mississippi | G | 1,13,23 | b | 1,5 | SZCDC |
|  | *Escherichia coli* |  |  |  |  | SZCDC |
|  | *Shigella* |  |  |  |  | SZCDC |
|  | *Proteus mirabilis* |  |  |  |  | SZCDC |

Table S2. Hybridization oligonucleotides probe sequences for specific *Salmonella* O and H antigens and the *ssaR* gene

| Target Antigen  Serogroup/Factor | Probe sequence (5’→3’) |
| --- | --- |
| A | F: GTGGCAGGGCGCTACGAACAATCCTAACGACTCTGTCTTCTCGTTCGTGACGTTATCTGGCATTGTTGGTTGTGCTGCAA |
|  | R: CCGAACCTGGCAACATACCATTAGATGTGAGATTGGATCTTGCTGGGC |
| B | F: GTGGCAGGGCGCTACGAACAATCCTAACGACTCTAGCTGCTCGTTCGTGACGGCAAATGTTATAAAACCATTAAAGCTTCTTGATTTGG |
|  | R: CAATAAAATATCGGGCGGATATCTTTTTAAATACAGTGAGATTGGATCTTGCTGGGC |
| C_1_ | F: GTGGCAGGGCGCTACGAACAATCCTAACGACTCTAGCTGCTTGTTCGTGACGTATTACTTGTGCTTGGTGCCATTCTATCATTG |
|  | R: CCTTTGTCACATTATTTATCAGATATAATTTCTTCCGTGAGATTGGATCTTGCTGGGC |
| C_2_-C_3_ | F: GTGGCAGGGCGCTACGAACAATCCTAACTACTCTCGCTGCTCGTTCGGGACGGGAACATCCAGACGTTTGTTTTATAAATTTACG |
|  | R: TTTAGAACATGTTTACGGTGAGAGGGATAAAGCAGTGAGATTGGATCTTGCTGGG |
| D1 | F: GTGGCAGGGCGCTACGAACAATCCTAACTACTCTTGCTGCTAGTTCGTGTCGCGAGTTTATATGCATATACTAAACAAAAAGCAAATGAAC |
|  | R: TCGCCGCCGCCATTATAGATAAAGTTTGTGAGATTGGATCTTGCTGGGC |
| E | F: GTGGCAGGGCGCTACGAACAATCCTAACGACTCTGGCTGCTCGTTCGTGACGGGTGCTGATTTAACCGGGTATTATTTATTAGTAG |
|  | R: TACAGTTGATGGGAGTATTAAATCTTGCTGAGCTAGTGAGATTGGATCTTGCTGGGC |
| a | F: GTGGCAGGGCGCTACGAACAATCCTATCCGTTCTTTATCGCTCAGCCTTCATCGGTCACGCCTTCGGCTACATTAAGCACTAC |
|  | R: TGCACTTGATGGTGCTGGCCTCAAATGAGATTGGATCTTGCTGGGC |
| b | F: GTGGCAGGGCGCTACGAACAATCCTATCGGTCCTTCATGGCTCAGTCTTCACCGGAAGTGTACAGGATGCCTATACGCCAAAAGG |
|  | R: TACCGCTGTTACCAGAGATGTTACCACCTATATGAGATTGGATCTTGCTGGGC |
| c | F: GTGGCAGGGCGCTACGAACAATCCTATCGGTCCTTCATGGCTCAGTCTTCACCGGATGCCTATACGCCAAAAGGTACCGCTGTTA |
|  | R: CCAGAGATGTTACCACCTATAAAAATGGTGGTGAGATTGGATCTTGCTGGGC |
| d | F: GTGGCAGGGCGCTACGAACAATCCTATCGGTCCTTCATCGCTCAGCCTTCACCGGGCCACTTATGATGAAACTACAAAGAAAGTTAAT |
|  | R: ATTGATACGACTGATAAAACTCCGTTGGC TGAGATTGGATCTTGCTGGGC |
| (e,n,z_15_) | F: GTGGCAGGGCGCTACGAACAATCCTAACGACTCTGGCTGCTCGTTCGTGACGTAAGGATGGTAAATATTATGCCACTGTAGGTGG |
|  | R: TTATACCGATGCGGGGGATACTGCCAATGAGATTGGATCTTGCTGGGC |
| i | F: GTGGCAGGGCGCTACGAACAATCCTAACTACTCTCGCTGCTCGTTCGGGACGCTGGTAAAGATGGCTATTATGAAGTTTCCGTTG |
|  | R: ATAAGACGAACGGTGAGGTGACTCTTGTGAGATTGGATCTTGCTGGGC |
| v | F: GTGGCAGGGCGCTACGAACAATCCTAACGACTCTAGCTTCTCGTTAGTGACGAAGTTAGTGTTGCAGATGATGGCACTGTTAC |
|  | R: AATGCCGACAACCACGAAAGTGACAGTGAGATTGGATCTTGCTGGGC |
| (g,m) | F: GTGGCAGGGCGCTACGAACAATCCTAACTACTCTTGCTGCTAGTTCGTGTCGTTAACTGTCGCTGATATTGCCACTGGCG |
|  | R: CGACGGATGTTAATGCTGCTACCTTACATGAGATTGGATCTTGCTGGGC |
| (z_4_,z_23_) | F: GTGGCAGGGCGCTACGAACAATCCTATCGGTCCTTCATCGCTCAGCCTTCACCGGGGTAGTTGGTGATGTAAAAATTGCGGCAGCT |
|  | R: GATTTCGATAACGCAAAAACAACTGGTGTGAGATTGGATCTTGCTGGGC |
| (e,h) | F: GTGGCAGGGCGCTACGAACAATCCTATCGGTGCTTCATAGCTCAGCCTTCACCGGAATCCCACGGCAACAGGCGATTCCTT |
|  | R: GTCTGCTACGCTTTCGTTTAAAGATGGTAAGTATTGAGATTGGATCTTGCTGGGC |
| (f,g) | F: GTGGCAGGGCGCTACGAACAATCCTATCCGTTCTTTATCGCTCAGCCTTCATCGGTGACGATGCGGAAAATAACACTGCGGTT |
|  | R: GACCTCTTTAAGACCACTAAATCTACTGCTGGTGAGATTGGATCTTGCTGGGC |
| (f,g,s/g,s,t) | F: GTGGCAGGGCGCTACGAACAATCCTATCGCTCCTTCATAGCTCAGACTTCATCGGGATATTAATTCAGGTGCTGTAGTAACTGATGATG |
|  | R: CAGCACCGGATAAAGTATATGTAAATGCAGCAATGAGATTGGATCTTGCTGGGC |
| r | F: GTGGCAGGGCGCTACGAACAATCCTAACGACTCTAGCTGCTTGTTCGTGACGGATGGAAAAGTCACTTTAACTGGCACACC |
|  | R: AACAGGACCAATTACTGCTGGCTTCCTGAGATTGGATCTTGCTGGGC |
| (1,2) | F: GTGGCAGGGCGCTACGAACAATCCTACGGTGACGCCCTTGGAAGGTTGTATACCATGATGCAGCTATTAAAGCGGCTACGGGT |
|  | R: GGTACGAATGGTACGGCTTCTGTAACTGAGATTGGATCTTGCTGGGC |
| (1,5) | F: GTGGCAGGGCGCTACGAACAATCCTACGGTGACGCCCTTGGAAGGTTGTATACCCGGCYAYDGGTGGTACGACTGGTA |
|  | R: CGGCTKCTGTAACSGGTRCRGTTAAATTTGATGAGATTGGATCTTGCTGGGC |
| (1,6) | F: GTGGCAGGGCGCTACGAACAATCCTACGGTAAGGCCATTGGCACGTTGTATACCAAAGCGGCTATAGGTGGTACGCTTGGCA |
|  | R: CGGCTTCTGTAACCGGTGGTACAGTGAGATTGGATCTTGCTGGGC |
| (1,7) | F: GTGGCAGGGCGCTACGAACAATCCTACGGTGAGGCCATTGGCAGGTTGTATACCCGGCTACGGGTGGTACGAATGGT |
|  | R: GCACCTAGTGTAACAGGTAGTGCGGTTAAATTTTGAGATTGGATCTTGCTGGGC |
| z_6_ | F: GTGGCAGGGCGCTACGAACAATCCTACGGTAAGGCCATTGGCACGTTGTATACCTTAAATCTGGCGGGATTACAGACCCAG |
| D2  *ssaR* | R: AAATTGCTGCTGCCCAGGTTGTCAAAATTGAGATTGGATCTTGCTGGGC  F: GTGGCAGGGCGCTACGAACAATCCTACGGTGAGGCCATTGGCAGGTTGTATACCAGGCTTTCTGATATTTATATATGATTTCTTATC  R: AGGGAAAAACACATAAGATTGTTTTTTGCACTGAGATTGGATCTTGCTGGGC  F: GTGGCAGGGCGCTACGAACAATCCTATCGGTCCTTTATCGCTCACCCTTCACCGG TTGCTCATATTAATTCCGGCATTTACGGTGAGTC  R: AGTTGACGCAGGCATTTCGGATTGGATTACTGAGATTGGATCTTGCTGGGC |
|  |  |

Table S3. Sequence of universal primers for LATE-PCR and fluorescent detection probes for melting curve analysis

| Primer/Probe | Sequence (5’→3’) |
| --- | --- |
| Universal primer-F  Universal primer-R | GTGGCAGGGCGCTACGAACAAT  GCCCAGCAAGATCCAATCTCA |
| Detection probe ROX | ROX-ACGACTCTGGCTGCTCGTTCGTGACG-BHQ |
| Detection probe FAM | FAM-TCGGTCCTTCATCGCTCAGCCTTCACCGG-BHQ |
| Detection probe Cy5 | Cy5-CGGTGAGGCCCTTGGCAGGTTGGTATCACCC-BHQ |

Table S4. Limit of identification values of each *Salmonella* serovar in the MLMA assay

| No. |  | *Salmonella* serotype | DNA concentration (ng/uL) |
| --- | --- | --- | --- |
| 1 |  | Typhimurium | 1.20 |
| 2 |  | Enteritidis | 1.20 |
| 3 |  | Paratyphi A | 1.56 |
| 4 |  | Typhi | 1.33 |
| 5 |  | Stanley | 1.32 |
| 6 |  | London | 1.20 |
| 7 |  | Derby | 1.22 |
| 8 |  | Senftenberg | 1.32 |
| 9 |  | Agona | 1.29 |
| 10 |  | Weltevreden | 1.45 |
| 11 |  | Anatum | 1.43 |
| 12 |  | Choleraesuis | 1.23 |
| 13 |  | Infantis | 1.45 |
| 14 |  | Muenchen | 1.52 |
| 15 |  | Braenderup | 1.34 |
| 16 |  | Papuana | 1.43 |
| 17 |  | Chennai | 1.23 |
| 18 |  | Rissen | 1.20 |
| 19 |  | Fillmore | 1.23 |
| 20 |  | Virchow | 1.54 |
| 21 |  | Saintpaul | 1.44 |
| 22 |  | Litchfield | 1.21 |
| 23 |  | Corvallis | 1.20 |
| 24 |  | Chester | 1.43 |
| 25 |  | Heidelberg | 1.34 |
| 26 |  | Give | 1.42 |
| 27 |  | Lagos | 1.22 |
| 28 |  | Essen | 1.35 |
| 29 |  | Nigeria | 1.44 |
| 30 |  | Paratyphi B | 1.45 |

Table S5. Reproducibility of designated melting temperature (Tm) tags for each gene target in the MLMA assay

| Antigen serogroup/  factor | Concentration  (bacilli/mL) | Intra-assay reproducibility | | |  | Inter-assay reproducibility | | |
| --- | --- | --- | --- | --- | --- | --- | --- | --- |
|  |  | Mean Tm (°C ) | SD | CV  % |  | Mean Tm (°C ) | SD | CV  % |
| O:3（E） | 2×10^10^ | 74.5 | 0.00 | 0.00 |  | 74.5 | 0.00 | 0.00 |
|  | 2×10^7^ | 74.5 | 0.00 | 0.00 |  | 74.5 | 0.00 | 0.00 |
| O:4（B） | 2×10^10^ | 70.5 | 0.00 | 0.00 |  | 70.5 | 0.00 | 0.00 |
|  | 2×10^7^ | 70.5 | 0.00 | 0.00 |  | 70.5 | 0.00 | 0.00 |
| O:8（C_2_-C_3_） | 2×10^10^ | 59 | 0.00 | 0.00 |  | 58.7 | 0.24 | 0.41 |
|  | 2×10^7^ | 58.7 | 0.24 | 0.40 |  | 58.7 | 0.24 | 0.41 |
| O:7（C_1_） | 2×10^10^ | 64 | 0.00 | 0.00 |  | 64 | 0.00 | 0.00 |
|  | 2×10^7^ | 64 | 0.00 | 0.00 |  | 64 | 0.00 | 0.00 |
| O:2（A） | 2×10^10^ | 67 | 0.00 | 0.00 |  | 67.3 | 0.29 | 0.43 |
|  | 2×10^7^ | 67.5 | 0.00 | 0.00 |  | 67.2 | 0.24 | 0.36 |
| O:9（D） | 2×10^10^ | 54.5 | 0.00 | 0.00 |  | 54.5 | 0.00 | 0.00 |
|  | 2×10^7^ | 55 | 0.00 | 0.00 |  | 54.5 | 0.00 | 0.00 |
| A | 2×10^10^ | 58.5 | 0.00 | 0.00 |  | 58.5 | 0.00 | 0.00 |
|  | 2×10^7^ | 58 | 0.00 | 0.00 |  | 58.5 | 0.00 | 0.00 |
| B | 2×10^10^ | 62 | 0.00 | 0.00 |  | 62.2 | 0.24 | 0.39 |
|  | 2×10^7^ | 62.5 | 0.00 | 0.00 |  | 62.3 | 0.29 | 0.46 |
| C | 2×10^10^ | 52.5 | 0.00 | 0.00 |  | 52.5 | 0.00 | 0.00 |
|  | 2×10^7^ | 52.5 | 0.00 | 0.00 |  | 52.5 | 0.00 | 0.00 |
| D1 | 2×10^10^ | 75.8 | 0.24 | 0.32 |  | 75.8 | 0.29 | 0.38 |
|  | 2×10^7^ | 75.3 | 0.27 | 0.36 |  | 75.7 | 0.24 | 0.32 |
| (1,2) | 2×10^10^ | 55.8 | 0.29 | 0.52 |  | 55.8 | 0.29 | 0.52 |
|  | 2×10^7^ | 55.3 | 0.27 | 0.49 |  | 55.3 | 0.29 | 0.52 |
| (1,7) | 2×10^10^ | 63.5 | 0.00 | 0.00 |  | 63.7 | 0.24 | 0.38 |
|  | 2×10^7^ | 63 | 0.00 | 0.00 |  | 62.3 | 0.27 | 0.43 |
| z_6_ | 2×10^10^ | 50.5 | 0.00 | 0.00 |  | 50.5 | 0.00 | 0.00 |
|  | 2×10^7^ | 51 | 0.00 | 0.00 |  | 50.5 | 0.00 | 0.00 |
| (e,n,x/e,n,z_15_) | 2×10^10^ | 74 | 0.00 | 0.00 |  | 74 | 0.00 | 0.00 |
|  | 2×10^7^ | 74 | 0.00 | 0.00 |  | 74 | 0.00 | 0.00 |
| r | 2×10^10^ | 62.2 | 0.24 | 0.39 |  | 62.3 | 0.24 | 0.39 |
|  | 2×10^7^ | 62.3 | 0.29 | 0.47 |  | 62.2 | 0.29 | 0.47 |
| i | 2×10^10^ | 59 | 0.00 | 0.00 |  | 59.5 | 0.00 | 0.00 |
|  | 2×10^7^ | 59.5 | 0.00 | 0.00 |  | 59.5 | 0.00 | 0.00 |
| v | 2×10^10^ | 57 | 0.00 | 0.00 |  | 57 | 0.00 | 0.00 |
|  | 2×10^7^ | 57 | 0.00 | 0.00 |  | 57 | 0.00 | 0.00 |
| (g,m) | 2×10^10^ | 54 | 0.00 | 0.00 |  | 54.5 | 0.00 | 0.00 |
|  | 2×10^7^ | 54.5 | 0.00 | 0.00 |  | 54.5 | 0.00 | 0.00 |
| (z_4_,z_23­­_) | 2×10^10^ | 75.5 | 0.00 | 0.00 |  | 75.3 | 0.29 | 0.39 |
|  | 2×10^7^ | 75 | 0.00 | 0.00 |  | 75.2 | 0.24 | 0.32 |
| (e,h) | 2×10^10^ | 64.3 | 0.29 | 0.45 |  | 63.8 | 0.29 | 0.45 |
|  | 2×10^7^ | 63.8 | 0.29 | 0.45 |  | 63.7 | 0.24 | 0.38 |
| (f,g) | 2×10^10^ | 58.8 | 0.29 | 0.49 |  | 58.3 | 0.29 | 0.50 |
|  | 2×10^7^ | 58.2 | 0.24 | 0.41 |  | 58.2 | 0.24 | 0.41 |
| (f,g,s/g,s,t) | 2×10^10^ | 52 | 0.00 | 0.00 |  | 51 | 0.50 | 0.98 |
|  | 2×10^7^ | 51.5 | 0.00 | 0.00 |  | 51.2 | 0.24 | 0.47 |
| (1,5) | 2×10^10^ | 57 | 0.00 | 0.00 |  | 57 | 0.00 | 0.00 |
|  | 2×10^7^ | 57 | 0.00 | 0.00 |  | 57 | 0.00 | 0.00 |
| (1,6) | 2×10^10^ | 50.5 | 0.00 | 0.00 |  | 50.2 | 0.29 | 0.58 |
|  | 2×10^7^ | 50 | 0.00 | 0.00 |  | 50.3 | 0.29 | 0.58 |
|  | 2×10^10^ | 66 | 0.00 | 0.00 |  | 66 | 0.00 | 0.00 |
| D2  *ssaR* | 2×10^7^  2×10^10^  2×10^7^ | 62  66  66 | 0.00  0.00  0.00 | 0.00  0.00  0.00 |  | 63  66  66 | 0.00  0.00  0.00 | 0.00  0.00  0.00 |

Table S6. *Salmonella* serovar identification by MLMA assay and conventional serotyping among clinical isolates (n=383) from the multicenter study

| No. | *Salmonella* serovars | Number of isolates | | kappa value |
| --- | --- | --- | --- | --- |
|  |  | MLMA | Conventional  serotyping |  |
| 1 | Typhimurium | 136 | 136 | 1 |
| 2 | Enteritidis | 93 | 93 | 1 |
| 3 | Paratyphi A | 1 | 1 | 1 |
| 4 | Typhi | 3 | 3 | 1 |
| 5 | Stanley | 14 | 14 | 1 |
| 6 | London | 34 | 34 | 1 |
| 7 | Derby | 15 | 15 | 1 |
| 8 | Senftenberg | 2 | 2 | 1 |
| 9 | Agona | 6 | 6 | 1 |
| 10 | Weltevreden | 10 | 10 | 1 |
| 11 | Anatum | 2 | 2 | 1 |
| 12 | Choleraesuis | 1 | 1 | 1 |
| 13 | Infantis | 3 | 3 | 1 |
| 14 | Muenchen | 1 | 1 | 1 |
| 15 | Braenderup | 1 | 1 | 1 |
| 16 | Papuana | 3 | 3 | 1 |
| 17 | Chennai | 14 | 14 | 1 |
| 18 | Rissen | 14 | 14 | 1 |
| 19 | Fillmore | 6 | 6 | 1 |
| 20 | Virchow | 1 | 1 | 1 |
| 21 | Saintpaul | 1 | 1 | 1 |
| 22 | Litchfield | 4 | 4 | 1 |
| 23 | Corvallis | 1 | 1 | 1 |
| 24 | Chester | 4 | 4 | 1 |
| 25 | Heidelberg | 1 | 1 | 1 |
| 26 | Give | 1 | 1 | 1 |
| 27 | Essen | 3 | 3 | 1 |
| 28 | Nigeria | 1 | 1 | 1 |
| 29 | Paratyphi B | 7 | 7 | 1 |
|  |  |  |  |  |
